# Efficacy and Safety of Thromboprophylaxis Post-Orthopedic Surgery

**DOI:** 10.7759/cureus.19691

**Published:** 2021-11-18

**Authors:** Khalid Alsheikh, Ahmed Hilabi, Abdulaziz Aleid, Khalid G Alharbi, Hussam S Alangari, Mohammed Alkhamis, Faisal Alzahrani, Wedad AlMadani

**Affiliations:** 1 Department of Orthopedics, College of Medicine, King Saud bin Abdulaziz University for Health Sciences, Riyadh, SAU; 2 Division of Orthopedic Surgery, King Abdulaziz Medical City, Ministry of National Guard–Health Affairs, Riyadh, SAU; 3 Department of Orthopedics, King Abdullah International Medical Research Center, Riyadh, SAU; 4 Department of Orthopedics, King Abdulaziz Medical City, Ministry of National Guard–Health Affairs, Riyadh, SAU; 5 Department of Epidemiology and Public Health, General Authority for Statistics, Ministry of Economy and Planning, Riyadh, SAU

**Keywords:** vte prophylaxis, meta-analysis, major orthopedic surgery, safety outcomes, thromboprophylaxis

## Abstract

Given the high risk of venous thromboembolism (VTE) post-orthopedic surgery and the vital role of thromboprophylaxis in preventing VTEs, this meta-analysis aimed to assess the efficacy of thromboprophylaxis post major orthopedic surgery and the relevant safety measures.

In this review, we conducted a computer-aided search of Google Scholar, PubMed, CINAHL, Cochrane, Medline, and EMBASE databases. We included all published randomized clinical trials (RCTs) that utilized enoxaparin, fondaparinux, dabigatran, rivaroxaban, apixaban, and aspirin for VTE prophylaxis in patients undergoing total hip arthroplasty (THA), hip fracture surgery, and total knee arthroplasty (TKA) based on primary and secondary outcomes. The Cochrane Collaboration tool was used to evaluate the risk of bias. All statistical analyses were performed using Review Manager Software.

A total of 23 RCTs were included with a total sample of 48,424 patients and an overall low risk of bias. The efficacy of enoxaparin in preventing VTEs in the TKA group was significantly better than fondaparinux. In the THA group, the efficacy of enoxaparin was significantly better than apixaban. The efficacies of fondaparinux, dabigatran, rivaroxaban, apixaban, and aspirin were comparable to that of enoxaparin in reducing VTE-associated mortality, major bleeding, and adverse events. In conclusion, we found that all included drugs were non-inferior to enoxaparin in VTE-associated mortality, major bleeding, and adverse events.

## Introduction and background

Venous thromboembolism (VTE) is a general term that includes the formation of a deep vein thrombus (DVT) in the legs, pelvis, or arms, which can lead the thrombus to dislodge and travel to the lungs, producing a pulmonary embolism (PE) or a cerebrovascular event in patients with patent foramen ovale (PFO) [1]. VTE affects 1 to 2 per 1,000 people each year, making it the third most prevalent cardiovascular disorder [2]. Annually, between 300,000 and 600,000 individuals are affected by VTE in the United States alone [3]. Risk factors associated with VTE include hereditary thrombophilia, obesity, oral contraceptive pills, fractures, and malignancy [4]. Another well-known risk factor for developing VTE is major surgeries, including orthopedic surgery, abdominal surgery, and pelvic surgery [5].

Major orthopedic surgery patients are more vulnerable to VTE than other surgeries, attributed mainly to prothrombotic processes such as coagulation cascade activation from tissue and bone injury, venous injuries, heat generation due to cement polymerization, reduced venous emptying intra- or post-surgery, and immobilization [6]. Recently, there has been a rise in the frequency of major surgeries such as total hip arthroplasty (THA), pelvic fracture surgery (PFS), hip fracture surgery (HFS), and total knee arthroplasty (TKA) [6,7]. Among the elderly, the most common procedures performed in the United States are joint replacement surgeries [7]. Moreover, approximately 4.7% of patients who undergo major orthopedic interventions without prophylaxis experience symptomatic VTE [8]. The incidence of fatal PE in patients using thromboprophylaxis has reduced from 3-7% to 0.1% in 90 days after surgery [9]. The pathophysiology behind these procedures that makes the patients prone to VTE relies on several factors such as manipulating the extremity, the use of a tourniquet, immobilization, and the use of polymethylmethacrylate (PMMA) cement [6]. Therefore, managing the risk of VT among patients who undergo major orthopedic interventions is vital [10].

Currently, several pharmacological thromboprophylaxis drugs are available, including unfractionated heparin (UFH), low-molecular-weight heparin (LMWH), direct inhibitors of factor Xa, aspirin, and vitamin K antagonists [10]. The most commonly used guidelines for thromboprophylaxis in orthopedic surgery include The American College of Chest Physicians (ACCP) guidelines (2012), the Scottish Intercollegiate Guidelines Network (SIGN) (2015), and the American Academy of Orthopedic Surgeons (2011) [11,12]. The ACCP guidelines suggest administering any of the following drugs to patients undergoing TKA or THA: LMWH, low-dose UFH, rivaroxaban, apixaban, fondaparinux, dabigatran, adjusted-dose vitamin K antagonists, and aspirin [13]. However, for patients undergoing HFS, the ACCP guidelines recommend administering either LMWH, low-dose UFH, adjusted-dose vitamin K antagonists, fondaparinux, or aspirin [13]. Even though all the previously mentioned drugs can be used, some studies have shown a preference for one drug over another. For example, aspirin, an inexpensive anti-platelet drug, shows no difference in its effect compared with rivaroxaban in patients undergoing TKA or THA [14]. Oral direct factor Xa inhibitors including rivaroxaban, dabigatran, and apixaban have the same VTE events compared with patients taking LMWH in orthopedic surgery [15-22]. On the other hand, rivaroxaban has been shown to be superior to enoxaparin [23-27]. In general, for patients scheduled to undergo major orthopedic surgery, guidelines suggest the extension of the thromboprophylaxis period up to 35 days in an outpatient setting [8]. Understanding the regimens, options, and dosages of thromboprophylaxis can reduce both mortality and morbidity among high-risk VTE patients. This study aims to investigate the efficacy and safety of thromboprophylaxis in orthopedic surgeries. It is essential to mention that the last known meta-analysis that conducted a pooled analysis was published in 2011 [28] and included agents that are almost obsolete in practice today. Given the high risk of VTEs post-orthopedic surgery, the vital role of thromboprophylaxis in preventing VTEs, and the need for a review article studying newer agents in orthopedic surgery, this meta-analysis aimed to evaluate the efficacy of thromboprophylaxis post-orthopedic surgery along with assessing the relevant safety measures, namely, major bleeding (defined by a drop in hemoglobin levels to ≥2 g/dL, or transfusion of two or more units of packed red blood cells, or bleeding into a critical organ, including bleeding into the operated joint), myocardial infarction, stroke, thrombocytopenia, and VTE-associated mortality.

## Review

Methodology

Search Strategy

A computer-aided search of Google Scholar, PubMed, CINAHL, Cochrane, Medline, and EMBASE databases from their inception to February 2021 was conducted. The following MeSH terms were used: major orthopedic surgery; orthopedic surgery; total knee replacement or arthroplasty or TKA; total hip replacement or arthroplasty or THA; shoulder arthroplasty, or long bone fractures or hip fractures or femur fractures or trauma or injury; all in variable combinations with venous thromboembolism, thromboembolism, and VTE, limited to one of the following agents at a time: aspirin or acetylsalicylic acid, rivaroxaban or BAY 59-7939, apixaban, enoxaparin, dabigatran or dabigatran etexilate, and fondaparinux. The electronic searches were supplemented by a manual search of relevant studies among the included articles. Included articles were screened on the basis of their title, abstract, and full text.

Inclusion Criteria

All studies with a randomized clinical trial design were included. Study subjects underwent major orthopedic surgery and were administered aspirin (acetylsalicylic acid), rivaroxaban (BAY 59-7939), apixaban, enoxaparin, dabigatran (dabigatran etexilate), and/or fondaparinux for prophylaxis. Studies exploring other agents and those using placebo were excluded.

Whenever trials included data for drugs not relevant to this meta-analysis, only data about the relevant agents were used. No gender restrictions were imposed. The primary outcomes screened were drug efficacy parameters such as VTE incidence, VTE-related mortality, major bleeding, and adverse events reported as total adverse events, any drug-related adverse event, or serious adverse events, such as myocardial infarction, stroke, or thrombocytopenia. Only articles published in the English language were included.

Study Selection

Three reviewers (AH, AA, KA) screened the titles and abstracts of all relevant publications. After screening all records, 23 trials met the inclusion criteria. Whenever clear inclusion criteria were not found, discussion and consensus between the authors were utilized to include or exclude studies.

Quality Assessment

Data extraction was done in parallel by two authors. An evidence table was generated for extraction to ensure consistency and accuracy. The table detailed the total number of participants, interventions, outcomes (in terms of efficacy and safety measurements), limitations, and the authors’ conclusions. The Cochrane Collaboration tool was used to evaluate the risk of bias (ROB) [13].

Data Synthesis and Analysis

Data were entered into Review Manager Software (RevMan version 5.3., Copenhagen: The Nordic Cochrane Centre, The Cochrane Collaboration, 2012) [13]. A random-effects model was devised using the DerSimonian and Laird approach to pool the results of the included studies. The I2 test and Cochrane’s Q test were used to assess heterogeneity. Statistical significance was defined as a p-value of <0.05.

Results

Selected Studies

**Figure 1:**
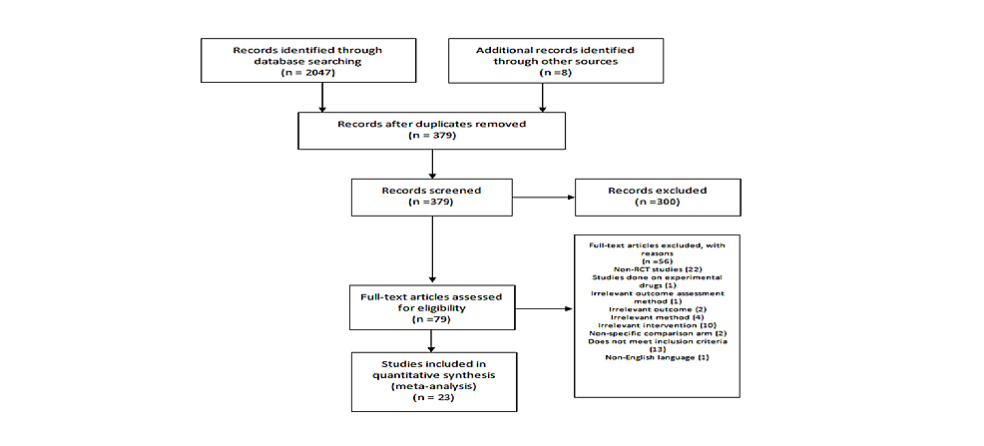
PRISMA flow diagram of the study protocol. PRISMA: Preferred Reporting Items for Systematic reviews and Meta-Analyses; RCT: randomized controlled trial

Figure 1 depicts the initial screening process, including the justification for exclusion. The total number of relevant randomized clinical trials (RCTs) identified during the initial search was 2,047. Overall, 23 RCTs were found to be eligible for inclusion. All included studies had a randomized double-blind design. The total number of participants across included RCTs was 48,424. The total number of patients with respect to each drug was as follows: enoxaparin: 19,867 patients, rivaroxaban: 12,307 patients, apixaban: 6,768 patients, dabigatran: 4,105 patients, fondaparinux: 3,668 patients, and aspirin: 1,709 patients. In total, 13 RCTs had a dual-arm design, allocating enrolled patients to receive a fixed dosage of either drug A or B. Eight RCTs had a dose-ranging design, allocating enrolled patients to different groups, each receiving a different dosage of either drug A or B. Two of the included RCTs had a multiple-arm design, allocating enrolled patients to receive a single dose of either drug A, B, or C. Most studies (n = 15) were multinational, and a few studies (n = 6) were local. Table 1 shows the characteristics of the included studies.

**Table 1:** Characteristics of included studies. THA: total hip arthroplasty/replacement; TKA: total knee arthroplasty/replacement; VTE: venous thromboembolism; HFS: hip fracture surgery; o.d.: once daily; b.i.d.: twice a day; q.d.: four times a day

Author, year, country	Procedure	Type of study	Intervention	Comparison	Outcomes reported	Limitations
Anderson et al., 2018, Canada [14]	THA/TKA	Double-blind, randomized controlled trial	Oral aspirin (81 mg o.d.)	Oral rivaroxaban (10 mg o.d.)	Aspirin and rivaroxaban exhibited comparable effects with respect to the prevention of symptomatic VTE with a similar safety profile	The authors did not calculate the absolute rates of VTE or bleeding complications associated with each of the two prophylaxis strategies. They were unable to ascertain the cause of bleeding as most bleeding events occurred shortly after randomization
Kim et al., 2016, South Korea [15]	THA	Single-center, prospective, randomized trial	Oral rivaroxaban (10 mg o.d.)	Enoxaparin (40 mg o.d.)	No difference in VTE risk between rivaroxaban and enoxaparin with a similar safety profile	This was a single-center study, and all 884 arthroplasties were performed by a single surgeon. The number of patients was inadequate to delineate the difference in efficacy. The study was underpowered to detect any difference in surgical complications
Lassen et al., 2010, Multinational [16]	THA	Double-blind, double-dummy, randomized clinical trial	Oral apixaban (2.5 mg b.i.d.)	Enoxaparin (40 mg o.d.)	Apixaban significantly decreased VTE risk in comparison to enoxaparin with a similar safety profile	None reported
Malhotra et al., 2017, India [17]	THA	Prospective, double-blind, double-dummy study	Oral dabigatran (220 mg o.d., starting with 110 mg, 1–4 hours post-operatively)	Subcutaneous enoxaparin (40 mg o.d., preoperatively)	Dabigatran and enoxaparin exhibited comparable efficacy and safety profiles	Insufficient sample size to compare both drugs
Erkisson et al., 2011, Multinational [18]	THA	Non-inferiority, double-blind, randomized trial	Oral dabigatran (220 mg o.d.)	Subcutaneous enoxaparin (40 mg) or placebo	Dabigatran was non-inferior to enoxaparin with a similar safety profile	None reported
Ginsberg et al., 2009, Multinational [19]	TKA	Double-blind, non-inferiority, active-controlled, randomized study	Oral dabigatran etexilate (150 or 200 mg o.d.)	Enoxaparin (30 mg daily)	Dabigatran dosing regimens exhibited inferior efficacy but similar safety (major bleeding)	None reported
Erkisson et al., 2007, Multinational [20]	TKA	Double-blind, non-inferior, active-controlled, randomized study	Dabigatran etexilate (150 or 220 mg o.d., starting with half-dose 1–4 hours post-operatively)	Enoxaparin (40 mg pre-operatively)	Dabigatran was non-inferior to enoxaparin with a similar safety profile	None reported
Eriksson et al., 2006, Multinational [21]	THA	Double-blind, comparator-controlled, double-dummy, randomized study	oral rivaroxaban (5, 10, 20, 30, or 40 mg)	Subcutaneous enoxaparin (40 mg)	Rivaroxaban and enoxaparin exhibited comparable efficacies and safety profiles post-THP	None reported
Turpie et al., 2005, Multinational [22]	TKA	Multicenter, parallel-group, double-dummy, double-blind study	Oral rivaroxaban (2.5, 5, 10, 20, and 30 mg b.i.d.)	Enoxaparin (30 mg b.i.d.)	Rivaroxaban and enoxaparin exhibited comparable efficacies and safety profiles	Few venograms were evaluable
Kakkar et al., 2008, Multinational [23]	THA	Multinational, double-dummy, double-blind, randomized trial	Oral rivaroxaban (10 mg o.d.)	Subcutaneous enoxaparin (40 mg o.d.)	Rivaroxaban exhibited higher efficacy compared to enoxaparin and placebo	Low sample size and high invalid venography rate of 25%
Eriksson et al., 2008, Multinational [24]	THA	Randomized, multinational, double-blind trial	Oral rivaroxaban (10 mg post-surgery)	Subcutaneous enoxaparin (40 mg o.d., pre-operatively)	Rivaroxaban exhibited higher efficacy compared to enoxaparin with a similar safety profile	Lower number of valid venograms that necessitated an increase in sample size
Lassen et al., 2008, Multinational [25]	TKA	Double-blind, multicenter, double-dummy, randomized trial	Rivaroxaban (10 mg o.d., 6–8 hours post-operatively)	Subcutaneous enoxaparin (40 mg, 12 hours pre-operatively)	Rivaroxaban exhibited higher thromboprophylactic efficacy compared to enoxaparin post-TKA, with comparable bleeding risk	Low number of valid venograms across the study
Turpie et al., 2009, Multinational [26]	TKA	Multicenter, randomized, double-blind phase 3 trial	Oral rivaroxaban (10 mg o.d., 6–8 hours post-operatively)	Subcutaneous enoxaparin (30 mg b.i.d., 12 hours pre-operatively)	Rivaroxaban exhibited higher efficacy compared to enoxaparin; there was no statistically different bleeding risk between the two arms	Higher number of inadequate venograms; surgical site bleeding was excluded from major bleeding
Eriksson et al., 2007, Europe [27]	THA	Randomized, open-label, active-comparator- controlled, dose-escalation study	Rivaroxaban (30 mg once daily or 2.5, 5, 10, 20, and 30 mg b.i.d., starting 6–8 hours post-operatively)	Enoxaparin (40 mg o.d. starting pre-operatively)	Incidences of major VTE decreased dose-dependently with increasing rivaroxaban dose. No rivaroxaban dose was significantly different from enoxaparin for major VTE	Bleeding risk could be over-reported because this was an open-label study, with wide confidence intervals. Study was not powered to detect differences between individual treatment groups
Lassen et al., 2010, Multinational [29]	TKA	Multicenter, double-blind, randomized, phase 3 study	Oral apixaban (2.5 mg b.i.d.)	Enoxaparin (40 mg o.d.)	Apixaban exhibited higher efficacy than enoxaparin with a similar safety profile	Higher rate of venograms that could not be assessed
Lassen et al., 2009, Multinational [30]	TKA	Double-blind, double-dummy, randomized clinical trial	Oral apixaban (2.5 mg b.i.d.)	Enoxaparin (30 mg o.d.)	Apixaban was not inferior to but exhibited lower bleeding risk than enoxaparin	Inability to obtain venograms that can be evaluated for all patients
Lassen et al., 2007, United States [31]	TKA	Randomized, eight-arm, parallel-group, multicenter, phase 2 trial	Oral Apixaban (2.5, 5, or 10 mg b.i.d., or 5, 10, or 20 mg q.d.)	Subcutaneous enoxaparin or oral warfarin	Apixaban exhibited lower efficacy compared to enoxaparin or warfarin	None reported
Eriksson et al., 2004, Europe [32]	THA/TKA	Double-blind, parallel-group, active-controlled, randomized study	Oral dabigatran (50 and 150 mg b.i.d., 300 mg o.d., or 225 mg b.i.d.)	Oral enoxaparin (40mg o.d.)	Dabigatran significantly reduced VTEs and major bleeding rates in their respective doses	None reported
Eriksson et al., 2006, Europe [33]	THA	Double-blind, prospective, comparator-controlled, double-dummy, randomized study	Oral rivaroxaban (2.5, 5, 10, 20, and 30 mg b.i.d.)	Enoxaparin (40 mg o.d.)	Rivaroxaban exhibited higher efficacy compared to enoxaparin for VTE prevention	The low rivaroxaban doses (<2.5 mg) were more efficacious but not evaluated
Bauer et al., 2001, Multinational [34]	TKA	Randomized, double-blind controlled trial	Subcutaneous fondaparinux (2.5 mg o.d.)	Enoxaparin (30 mg b.i.d.)	Fondaparinux exhibited significantly higher efficacy compared to enoxaparin; however, the fondaparinux group exhibited higher bleeding risk	VTE risk was lowered because patients received a therapeutic dose of anticoagulant and 20% patients received prophylaxis after termination of the study
Turpie et al., 2002, Multinational [35]	THA	Double-blind randomized clinical trial	Subcutaneous fondaparinux (2.5 mg o.d., post-operatively)	Subcutaneous Enoxaparin (40 mg b.i.d., post-operatively)	Fondaparinux exhibited lower efficacy compared to enoxaparin with a similar safety profile	Some patients were treated with therapeutic doses and more than a quarter of patients underwent prolonged prophylaxis
Lassen et al., 2002, Europe [36]	THA	Multicenter, randomized, double-blind trial	Fondaparinux (2.5 mg o.d., post-operatively)	Enoxaparin (40 mg o.d., pre-operatively)	Fondaparinux exhibited higher efficacy compared to enoxaparin with a similar bleeding risk	Some patients were treated with therapeutic dose, and more than a quarter of patients underwent prolonged prophylaxis
Eriksson et al., 2001, Multinational [37]	HFS	Randomized, double-blind, controlled trial	Fondaparinux (2.5 mg o.d.)	Enoxaparin (40 mg o.d.)	Fondaparinux exhibited higher efficacy compared to enoxaparin with a similar safety profile	Treatment duration might be too short for at-risk patients before it was discontinued

Risk of Bias

**Figure 2:**
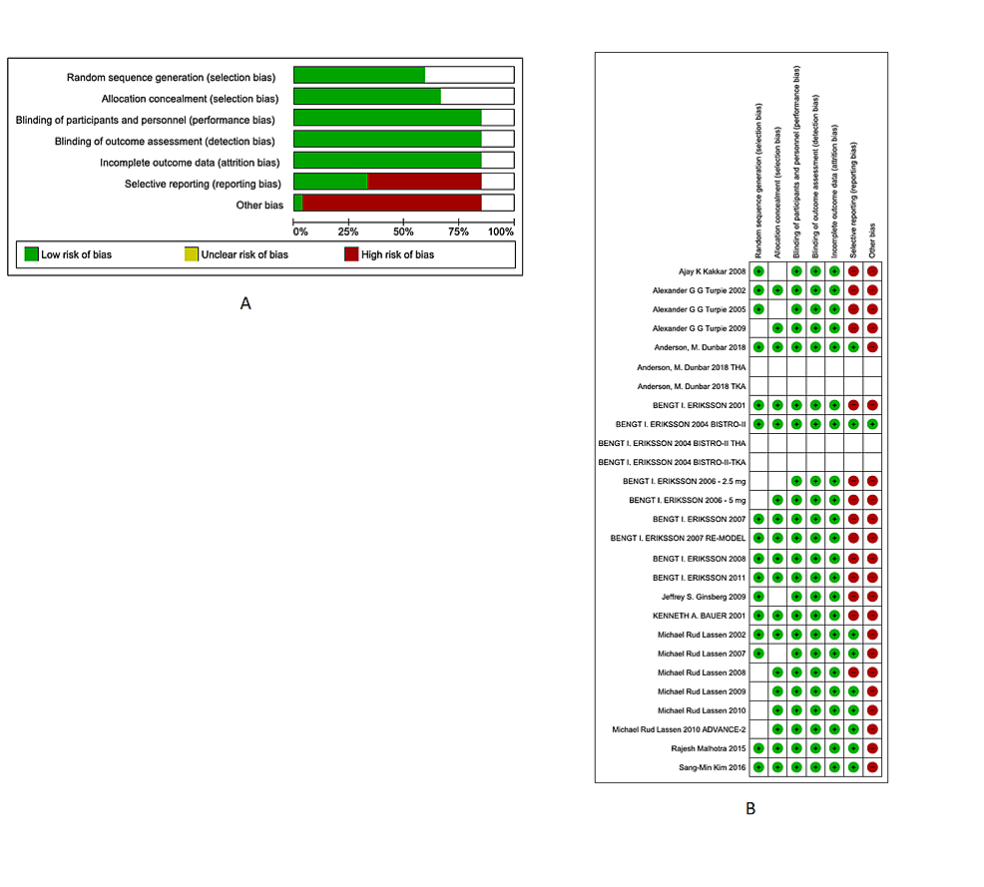
Overall ROB per bias item (A) and ROB items within each study (B). ROB: risk of bias

The Cochrane Collaboration tool was used to assess the overall ROB per bias item (Figure 2A). The overall quality of the studies was high, except selective reporting and other biases (being sponsored by and/or affiliated with a pharmaceutical company), which had an ROB of 60% and 95%, respectively. The remaining items, namely, incomplete outcome data bias, allocation concealment, random sequence, blinding of outcome assessment, and blinding of personnel and participants, were of high quality. Further details regarding ROB items within each study are shown in Figures 2A, 2B.

Primary Outcomes

**Figure 3:**
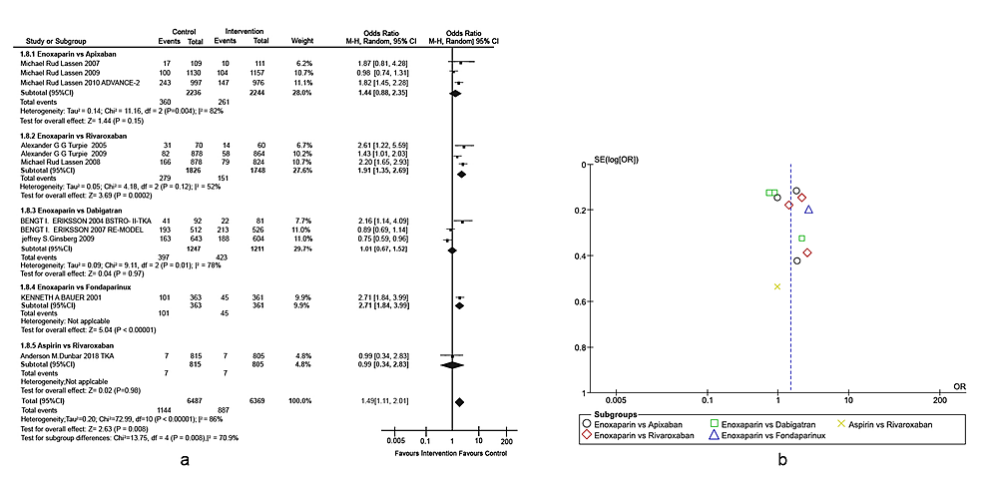
Comparison of VTE incidence in TKA. (A) Forest plot of different types of active comparators, reporting high heterogeneity. (B) Funnel plot of different types of active comparators, reporting high bias. VTE: venous thromboembolism; TKA: total knee arthroplasty

Prophylaxis of total knee arthroplasty venous thromboembolism: The primary outcome was VTE incidence in different types of active comparators, as presented in a forest plot (Figures 3A, 3B), detailing the TKA-associated risks. This outcome was defined as any form of PE or DVT that occurred during the treatment period. Enoxaparin exhibited significantly higher efficacy in VTE prevention of approximately five-fold higher than fondaparinux (Overall effect: Z = 5.04; p-value < 0.00001, 95% CI = [1.84, 3.99]). Other interventions were non-inferior to enoxaparin in reducing the incidence rates of VTE post-TKA. A heterogeneity of 86% was observed across studies, which was mainly attributed to the differences in the doses used by Lassen et al. [29-31]. In the subgroup of enoxaparin versus dabigatran, the study conducted by Eriksson et al. was the only one that included 50 mg of oral dabigatran, whereas the rest of the studies included 150 mg and 225 mg of oral dabigatran [32].

**Figure 4:**
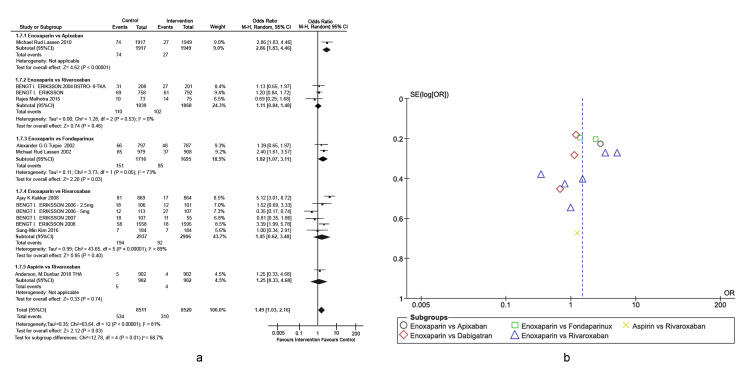
Comparison of VTE incidence in THA. (A) Forest plot of different types of active comparators. (B) Funnel plot of different types of active comparators, reporting high bias. VTE: venous thromboembolism; THA: total hip arthroplasty

Prophylaxis of total hip arthroplasty venous thromboembolism: The primary outcome was VTE incidence in different types of active comparators, as depicted in the forest plot (Figures 4A, 4B) detailing THA-associated risks. This outcome is defined as any form of PE or DVT that occurred during the treatment period. Enoxaparin exhibited four-fold higher efficacy in preventing VTEs compared to apixaban (overall effect: Z = 4.62, p-value < 0.00001, 95% CI = [1.83, 4.46]). Enoxaparin also exhibited significantly higher efficacy in preventing VTEs compared to rivaroxaban (overall effect: Z = 0.85, p-value < 0.00001, 95% CI = [0.62, 3.40]). Other interventions were non-inferior to enoxaparin in reducing the rates of VTEs post-THA. A heterogeneity of 81% was observed across the studies, which was primarily attributed to the studies conducted by Eriksson et al. (BISTRO-II) and Lassen et al. (ADVANCE-2) [16,33]. The study conducted by Eriksson et al. (2008) is the only study in which drugs were administered pre-operatively. In addition, rivaroxaban was administered in different doses [24].

Secondary Outcomes

**Figure 5:**
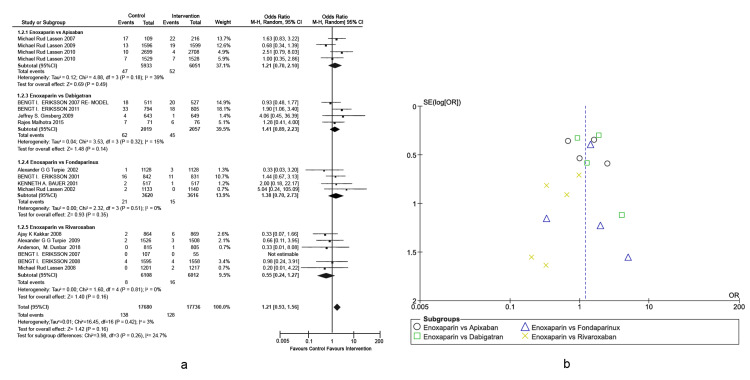
Comparison of VTE-associated mortality for patients undergoing THA, TKA, and HFS. (A) Forest plot of different types of active comparators. (B) Funnel plot of different types of active comparators. THA: total hip arthroplasty; TKA: total knee arthroplasty; HFS: hip fracture surgery

Venous thromboembolism-associated mortality: One of the secondary outcomes was VTE-associated mortality in the VTE prophylaxis medication groups, as shown in the forest plots (Figures 5A, 5B). VTE-associated mortality was defined as death due to a perioperative thrombotic event, excluding any other causes of non-survival. We observed that fondaparinux, dabigatran, rivaroxaban, apixaban, and aspirin were non-inferior to enoxaparin in reducing VTE-associated mortality rates. Heterogeneity performance across included studies was reported to be 3%.

Major Bleeding

**Figure 6:**
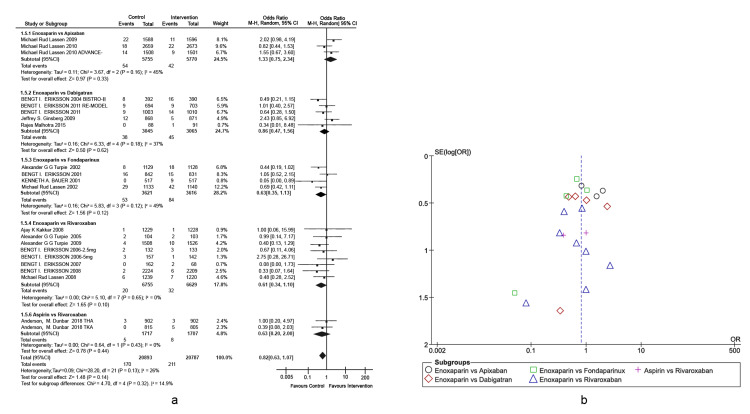
Comparison of major bleeding events for patients undergoing THA, TKA, and HFS. (A) Forest plot of different types of active comparators. (B) Funnel plot of different types of active comparators. THA: total hip arthroplasty; TKA: total knee arthroplasty; HFS: hip fracture surgery

Another important secondary outcome was major bleeding episodes in different medication groups, as shown in the forest plot in Figures 6A, 6B. Major bleeding, defined similarly across all included studies, is either a drop in hemoglobin levels of ≥2 g/dL, transfusion of two or more units of packed red blood cells, or bleeding into a critical organ, including bleeding into the operated joint, as well as the need for surgical intervention, lasting up to 11 days since the administration of prophylaxis. Even though this finding was non-significant, overall, enoxaparin showed fewer major bleeding episodes than fondaparinux, dabigatran, rivaroxaban, apixaban, and aspirin. Heterogeneity performance across included studies was reported to be 26%.

Adverse Events

**Figure 7:**
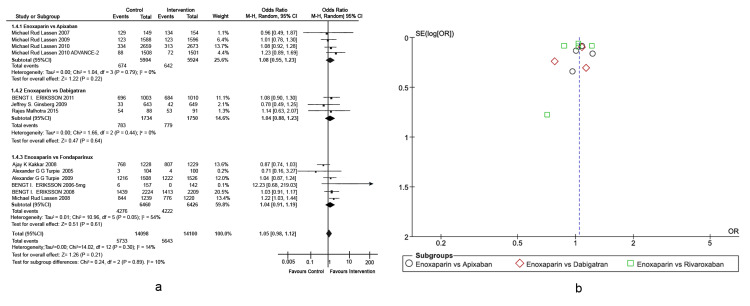
Comparison of total adverse events for patients undergoing THA, TKA, and HFS. (A) Forest plot of different types of active comparators. (B) Funnel plot of different types of active comparators. THA: total hip arthroplasty; TKA: total knee arthroplasty; HFS: hip fracture surgery

Adverse events are defined as unplanned occurrences that are detrimental to the patient and occur during the treatment period up to two days after treatment cessation unanimously by included RCTs, as shown in the forest plots in Figures 7A, 7B. Adverse events included events that were either drug-related side effects and/or any other serious events such as stroke, myocardial infarction, and pancytopenia. Overall, fondaparinux, dabigatran, rivaroxaban, apixaban, and aspirin proved to be non-inferior to enoxaparin in reducing the rates of adverse events. Heterogeneity performance across included studies was calculated to be 14%.

Discussion

Patients undergoing TKA, THA, or major orthopedic surgery for fractures exhibit a higher risk for VTEs unless they receive thromboprophylaxis. Therefore, the current guidelines for patients undergoing TKA involve using a prophylactic anticoagulant for at least 10 days post-TKA [2]. Our meta-analysis included data from 23 RCTs comparing different thromboprophylaxis regimens, including antiplatelets, such as aspirin, and anticoagulants, such as rivaroxaban, apixaban, dabigatran, enoxaparin, and fondaparinux either before or after TKA, THA, or any major orthopedic surgery for fractures. This meta-analysis showed that, in patients undergoing TKA, enoxaparin was more effective than fondaparinux in decreasing the risk of VTE. However, apixaban, rivaroxaban, and dabigatran were non-inferior to enoxaparin in decreasing VTE incidence in the same setting. In patients undergoing THA, enoxaparin was found to be more effective than rivaroxaban and apixaban in preventing VTE but was non-inferior to aspirin, dabigatran, and fondaparinux. However, this finding should be interpreted with caution due to the high heterogeneity reported in our analysis. Overall, we conclude that enoxaparin can be more effective or non-inferior to other drugs depending on the setting.

Furthermore, the enoxaparin group exhibited fewer major bleeding events than fondaparinux, though this finding was non-significant. The major bleeding rates for patients treated with enoxaparin were comparable to those seen in patients treated with apixaban, rivaroxaban, dabigatran, and aspirin. Therefore, we could not conclude that enoxaparin was better than any of the comparators to reduce major bleeding events in patients undergoing TKA, THA, or any other major orthopedic surgery.

Moreover, we observed that comparators were non-inferior to enoxaparin with respect to the incidence of adverse events. These findings need to be interpreted with caution because not all included RCTs reported the incidence of adverse events in their cohorts.

Our findings are consistent with a previous meta-analysis that evaluated the efficacy of aspirin against rivaroxaban and LMWH in patients undergoing TKA and THA [38]. In this meta-analysis, the authors demonstrated that aspirin was non-inferior to rivaroxaban and LMWH in reducing the risk of VTE and in the safety profile/incidence of adverse events. Our results were also similar to those of other extensive observation cohort studies [39,40]. Therefore, the current evidence supports that enoxaparin, fondaparinux, dabigatran, apixaban, rivaroxaban, and aspirin demonstrate comparable efficacy in VTE prophylaxis among patients who underwent THA, TKA, or any other major orthopedic surgery for fractures.

Many of the included RCTs eliminated some participants because their venogram results were suboptimal, which is a feature of many RCTs in this research field. As in the RECORD1 trial, 4,541 patients underwent randomization. Of these, 3,029 (67%) patients were included in the per-protocol analysis of primary efficacy. Patients who could not be assessed for efficacy were unlikely to yield unbalanced results because the majority of the included RCTs tried to overcome this limitation by increasing the number of recruited patients or performing sensitivity analysis to avoid underpowered studies or biased outcomes.

Our findings are based on a pooled analysis of 48,424 patients who underwent TKA, THA, or other major orthopedic surgery for fractures and were given prophylactic anticoagulant for VTE. To our knowledge, these results represent the first reported pooled analysis comparing rivaroxaban, apixaban, dabigatran, aspirin, fondaparinux, and enoxaparin for VTE prevention post-orthopedic surgery. The most notable strength of our meta-analysis is the large sample size and the focus on robust and high-quality RCTs. Furthermore, the definition of efficacy and safety outcomes was universally similar between included RCTs, which increases the objectiveness of the results and reduces inter- and intra-group differences.

However, this meta-analysis is not without limitations. Our meta-analysis assessed VTE prophylaxis in different patient settings with different medications, doses, and schedules. For example, 17 RCTs used a 40 mg dose of enoxaparin once daily as an intervention or for comparison, whereas six RCTs used a 30 mg dose of enoxaparin twice daily as prophylaxis for VTE. These differences cannot be prevented because the included RCTs used different medications and schedules of prophylactic treatment. Second, many included RCTs had an insufficient number of venograms that were accessible for interpretation. However, the authors of these RCTs tried to overcome this limitation by increasing the number of included patients at later stages. Third, RCTs assessing apixaban as a prophylactic treatment for VTE, especially the ADVANCE trials, were funded and supported by pharmaceutical companies who hold the patent for apixaban, which may be a source of bias in the treatment outcomes. Lastly, although most included RCTs used bilateral venograms to diagnose DVT, some RCTs used venograms on the leg, on which the operation was done, and some studies diagnosed DVT using only Doppler ultrasound.

## Conclusions

Although the overall effect of the drugs included in this study, namely, fondaparinux, dabigatran, rivaroxaban, apixaban, and aspirin, were non-inferior to enoxaparin in terms of VTE-associated mortality, major bleeding, and adverse events. In TKA VTE prophylaxis, enoxaparin efficacy was five-fold higher than fondaparinux. In TKA VTE prophylaxis, enoxaparin was more effective than apixaban and rivaroxaban with no inferiority to other drugs.
